# Evaluation of Protective Efficacy of Influenza Virus Like Particles Prepared from H5N1 Virus of Clade 2.2.1.2 in Chickens

**DOI:** 10.3390/vaccines9070715

**Published:** 2021-07-01

**Authors:** Mohamed H. El-Husseiny, Naglaa M. Hagag, Peter Pushko, Irina Tretyakova, Mahmoud M. Naguib, Abdel Satar Arafa

**Affiliations:** 1 Reference Laboratory for Veterinary Quality Control on Poultry Production (RLQP), Animal Health Research Institute (AHRI), Agriculture Research Center (ARC), Giza 12618, Egypt; naglaahagagahri@gmail.com (N.M.H.); mahmoud.naguib@imbim.uu.se (M.M.N.); abd.arafa@gmail.com (A.S.A.); 2 Medigen, Inc., Frederick, MD 21701, USA; ppushko@medigen-usa.com (P.P.); itretyakova@medigen-usa.com (I.T.)

**Keywords:** virus like particles, highly pathogenic avian influenza H5N1, clade 2.2.1.2, protection study

## Abstract

Highly pathogenic Avian Influenza (HPAI) viruses continue to cause severe economic losses in poultry species worldwide. HPAI virus of subtype H5N1 was reported in Egypt in 2006, and despite vaccination efforts, the virus has become endemic. The current study aims to evaluate the efficacy of a virus-like particle (VLP) based vaccine in vivo using specific pathogen-free (SPF) chickens. The vaccine was prepared from the HPAI H5N1 virus of clade 2.2.1.2 using the baculovirus expression system. The VLPs were quantitated and characterized, including electron microscopy. In addition, the protection level of the VLPs was evaluated by using two different regimens, including one dose and two-dose vaccinated groups, which gave up to 70% and 100% protection level, respectively. The results of this study emphasize the potential usefulness of the VLPs-based vaccine as an alternative vaccine candidate for the control of AIV infection in poultry.

## 1. Introduction

In early 2006, the highly pathogenic avian influenza (HPAI) virus of the H5N1 subtype was reported in Egypt and spread very rapidly within all sectors in the country, causing several outbreaks in different poultry species [1]. Since then, the virus has become endemic among poultry. In 2014/2015, a new cluster of HPAI H5N1 viruses within clade 2.2.1.2 emerged in Egypt and was associated with a high number of human cases [2]. The decision to use mass vaccination against HPAI in Egypt was made as an emergency measure based on the positive impact of such blanket vaccination in Vietnam and China [3].

Vaccination is the major prophylactic strategy to reduce diseases caused by an influenza virus infection [4,5]. Several recent studies focused on developing non-replicating virus-like particles (VLPs) as alternative influenza vaccines. Moreover, VLPs-based vaccines received significant attention for their potential promise in developing effective and safe vaccines against influenza viruses [6,7]. Commercialized prophylactic VLPs-based vaccines have been developed, including baculovirus-derived papillomavirus VLPs vaccines against cervical cancer and hepatitis B virus VLPs vaccines against chronic hepatitis B virus infection [8,9], which support the development of additional baculovirus-derived vaccines. Influenza VLPs are a new generation of egg-independent candidate vaccines that encode genes for the three influenza virus proteins, hemagglutinin (HA) and neuraminidase (NA), along with the viral core protein matrix (M1) [10,11]. However, the application of VLP-based vaccines against AIVs is still under optimization. Promising trials were reported for the HPAI H5N1 subtype VLPs-based vaccines based on multiple clades of H5N1 (clade 1; clade 2.2.1.1, and clade 2.3.2.1) and treated with beta-propiolactone in a ferret model [12]. Another trial was performed in BALB/c mice using HPAI H5N1 VLPs in a single dose regimen [13]. Moreover, HPAI H5N1 VLPS were tested in chicken experiments and formulated with a commercial adjuvant SEPPIC, Montanide 70/30, using a double dose [14]. However, none of those previous studies investigated viruses of clade 2.2.1.2.

In the current study, we aimed to evaluate the size, structure, and integrity of previously generated VLPs derived from the HPAIV H5N1 of clade 2.2.1.2 by using electron microscopy. Further, we evaluated the protection of the VLPs-based vaccine in SPF chickens without adjuvant by using two different regimens: one-dose vs. two-doses without using adjuvant.

## 2. Materials and Methods

### 2.1. VLPs Preparation and Characterization

The construction of VLPs was conducted as described in the previous study [11]. VLPs were based on a recombinant Baculovirus (rBV) expressing system using the HPAI H5N1 virus (A/chicken/Egypt/121/2012) [15]. In this study, we additionally explore the morphology and the size of the generated VLPs using Electron microscopy. VLP samples were adsorbed onto freshly discharged 400 mesh carbon parlodion-coated copper grids (Poly-Sciences, Warrington, PA USA). The grids were rinsed with buffer containing 20 mM Tris, pH 7.4, and 120 mM KCl and negatively stained with 1% phosphotungstic acid, then dried by aspiration. VLPs were visualized on a Hitachi H-7600 transmission electron microscope (Hitachi High Technologies America, Schaumburg, IL USA) operating at 80 kV and digitally captured with a CCD camera at 1 kx1 k resolution (Advanced Microscopy Techniques Corp., Danvers, MA, USA) [10].

### 2.2. Evaluation of the VLPs in SPF Chickens as a Candidate Vaccine

The experimental protocol of this study was evaluated and approved by the Review Board of the Animal Health Research Institute (AHRI) (AHRI-2020827). The animal experiment was performed at the animal facility unit at AHRI. A total of 40 SPF chicks were purchased from Kom Oshem Farm, El-Fayoum, Egypt, and were kept in Biosafety Level 3 isolators from the day of delivery till the end of the experiments with daily observation and care. All efforts were made during swabs and blood collection to minimize the suffering of the birds.

Four groups of ten 7-days old chicks each were separated at BSL3 isolators. The first group was vaccinated twice subcutaneously (S/C) at the neck fold; at one week old (7 days old) with the prepared VLPs without adjuvant and at 3 weeks after the first dose (28 days old) without adjuvant too. The two doses were adjusted to 2^9^ HA units. The second group was subjected to the VLPs vaccine via the same route with only one dose adjusted to 2^9^ HA units at 4 weeks (28 days old). The third group was considered as the negative control group and received only phosphate-buffered saline (PBS) in place of VLPs and no virus challenge, while the fourth group which was considered the positive control group, was challenged and not vaccinated. The chickens were wing-banded for identification, received feed and water ad libitum, and kept in BSL3 animal isolators in RLQP animal facilities. All groups except the negative control group were inoculated intranasally with 10^6^ EID50/0.1 mL of the homologous strain A/chicken/Egypt/121/2012 A/(H5N1) after 3 weeks of the booster dose in group 1 (at 49 days old), and 3 weeks after the only prime dose in group 2 (at 49 days old). The birds were observed daily for signs and mortality for 10 consecutive days post-challenge. Blood samples were collected from the wing vein on the same day of the first and second dose of VLPs administration in group one and on the same day of the only dose of VLPs administration in group 2. Moreover, blood samples were collected on the same day of challenge in both groups 1 and 2. A hemagglutination inhibition (HI) test was performed in V-bottom 96-well microtiter plates by using four hemagglutinating units (4 HAU) of the VLPs as antigen. Firstly, the antibodies from the VLP-immunized SPF chickens were subjected to a two-fold serial dilution with PBS, then the VLPs were added as antigen prior to the addition of the 1% chicken blood cells according to the OIE standard protocol [16].

## 3. Results

### 3.1. Morphology and Size of VLPs

**Figure 1:**
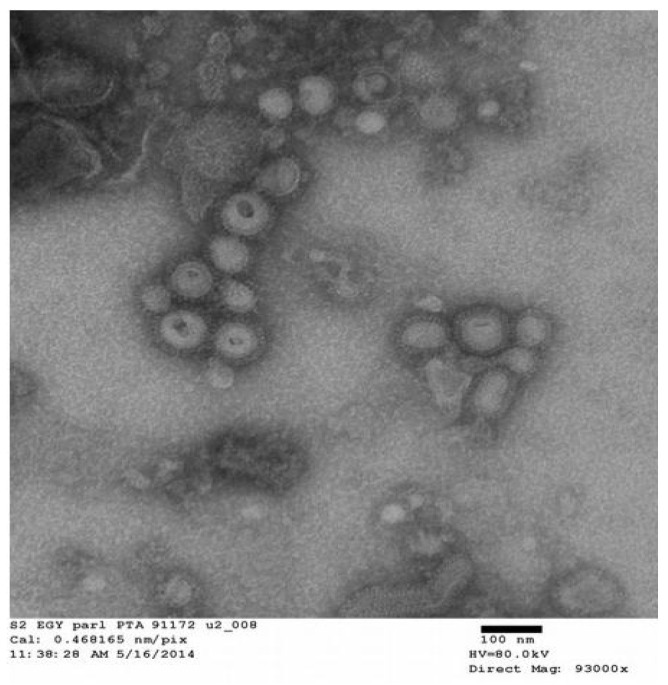
Negative stain transmission electron microscopy. For electron microscopy, VLPs were stained with 1% phosphotungstic acid showing the spherical influenza-like enveloped particles (VLPs).

The electron microscopic investigation of the negatively stained samples illustrated the presence of influenza A/chicken/Egypt/121/2012 A/(H5N1). The VLPs show a diameter of about 80–120 nm, which resemble the morphology and size of the influenza virus particles and were enveloped pleomorphic and spherical particles. The VLPs were associated frequently as groups resembling bead-like structures, with characteristics of influenza HA protein spikes with a lipid membrane on virions (Figure 1).

### 3.2. Immunogenicity in Chickens

**Figure 2:**
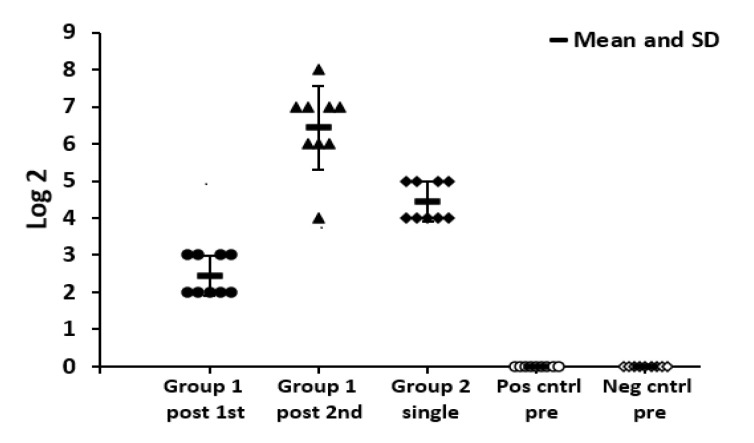
Chart for displaying the individual HI titers in log 2 after injection of the prepared VLPs without adjuvant. The chart shows the immune response of chickens in group-1 at 3-weeks after the injection of the first dose (Group1 post 1st), 3-weeks after second dose (Group1 post 2nd), and the immune response after 3 weeks by the injection of the single dose in group 2 (Group 2 single). Moreover, the positive control group pre-challenge (Pos cntrl pre) and negative control group pre-challenge (Neg cntrl pre). The figur shows the standard deviation (SD) and mean.

**Figure 3:**
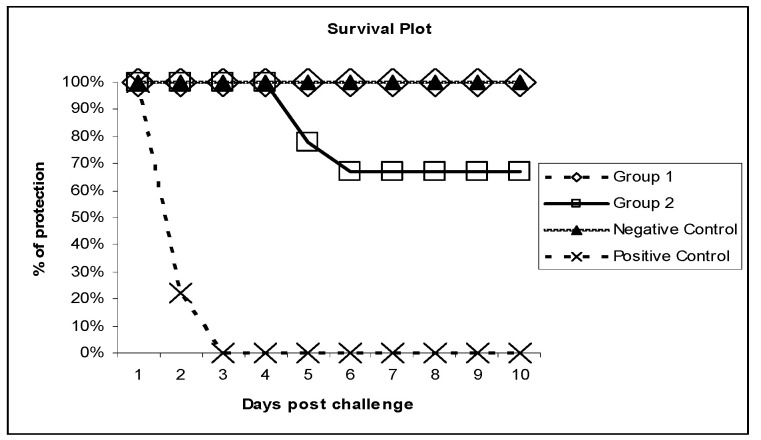
Survival plots indicating protection of H5N1 VLPs vaccinated chickens against HPAI H5N1 clade 2.2.1.2. The plot includes both groups of the current study, as well as the positive and negative control groups.

The evaluation of the immunogenicity of the VLPs was done in SPF chickens. No H5-specific serum antibody was detected in the chickens before vaccination during the evaluation study. After the prime dose, chickens in group-1 developed HI titers that increased after the booster dose. Moreover, in the second group, antibodies were detected after the single dose. However, in both groups, there was one chicken that lacked HI titer and died. The HI titer and mortality percent are summarized in Figure 2, Figure 3, and Table 1.

## 4. Discussion

In the present study, we characterized VLPs expressed in the baculovirus expression system (BEVS) from HPAI A/(H5N1) of recently emerged clade 2.2.1.2 [15] by using electron microscopy. Electron micrographs revealed spherical VLPs with a diameter of approximately 120 nm, which are similar in size to influenza VLPs comprised of an M1 protein core and a lipid envelope containing the HA protein [9,11,12,13]. BEVS VLPs are smaller than influenza VLPs prepared using Retrovirus Gag Protein core, which has a diameter of 150–200 nm. A protection level of up to 100% and 70% was found in group-1 (two doses of VLPs-based vaccine) and in group-2 (one dose of VLPs-based vaccine), respectively. The protection percent of the subcutaneous routes of the two dose regimen of the first group could be accepted as recommended by the 2015 OIE manual, but the one dose regimen gave lower protection.

Previous studies proved that the VLPs vaccination administrated via intranasal route could give 100% protection in mice and ferret against different AIV subtypes, including the HPAI H5N1 subtype [17,18]. To the best of our knowledge, all trials using the VLPs-based vaccine in poultry against AIV or other viruses as ND, IBV, IBDV were performed via an intramuscular or subcutaneous route of vaccination as used in our study [19,20]. Hence, further studies are required to determine the validity of the intranasal route for VLPs-based vaccines in poultry as in mice and ferret. Potentially, the intranasal route can enable mass vaccination, which is very important in the veterinary field vaccination.

Regarding the HI result after 3 weeks of the booster dose in group 1, we found that the mean titer was 2^6^ HIU which is above the cut-off of the protective level 40 HIU unit [21]. In this study, a one-shot regimen gave low protection (~70%), which might be due to an inherent difference in the immunogenicity of the HA proteins of the selected isolate for chickens which correlated to selection to evade the immune response [22]. A previous study for the H7N3 subtype revealed poor immunity and protection against the homologous strain [23]. This observation highlights the importance of highly immunogenic strain selection as a candidate vaccine seed. In the same context, the incomplete protection may be due to the post-translational modifications which differ in insect compared to mammalian species. This requires further experimental studies, including protein configuration folding, folding signals, and the chaperone requirements of the glycoprotein formation in insect cell Endoplasmic Reticulum and Golgi [24]. As a result of differences in post-translational patterns, the biological properties and/or the immunogenicity of the recombinant proteins might differ from native mammalian proteins [25]. However, escalating protection by this regimen can be achieved by using more immunogenic seed virus, a higher concentration of the VLPs construct in balance with the cost, and using a suitable adjuvant for proper longer and exaggerated action on the immune system of the birds [26].

From this study, we can summarize that the VLP vaccine is immunogenic and protective in chickens by the S/C route. The recombinant VLP manufacturing process is safe, making VLP a promising vaccine candidate that can have a great impact on the control of avian influenza virus in different poultry sectors, particularly if the cost of manufacturing can be minimized as previously proposed [27]. The low cost of the vaccine for veterinary use is very important to be available in the marketplace. The adjuvant cost usually represents more than 50% of the total cost of the commercial vaccines. Hence, one of our objectives in this research was to test VLPs candidates without adjuvant in order to minimize and reduce the cost of the innovative VLP vaccinal candidates. In addition, the cost could be minimized by using more than one AIV subtype on the same VLPs molecule, especially for the countries where more than one AIV subtype is circulating [28]. In addition, the optimization of low-cost purification methods has a positive impact on the total cost of the vaccine. Using a one shot regimen is considered the most significant challenge, which will greatly affect the cost and avoid the vaccination process stress and the post-vaccination reaction, in turn improving the health and welfare of animals and increasing poultry production in a cost-effective manner [29].

## 5. Conclusions

We conclude that the H5N1-VLPs-based vaccine can be recommended as a prototype candidate vaccine. Additional studies are needed to enhance the efficacy of this vaccine, including the addition of adjuvant and application of the one-dose regimen using a higher dose. In addition, the development of an intranasal vaccination route would allow the vaccine to be used for mass vaccination application. Our data suggest that an optimized H5N1-VLPs-based vaccine can be considered as an alternative vaccine strategy for effective AIV control.

## Figures and Tables

**Table 1:** Immunogenicity and protection in the two groups of chickens after vaccination by VLPs without adjuvant by using distinct vaccination regiments, including the HI geometric mean titer in comparison to controls.

Group.	Route	HI Geometric Mean Titer, log2, after 3wk of 1st Dose	HI geometric Mean Titer, log2, after 3wk of 2nd dose	Challenge Virus Titer	Mortality	Protection %
Group 1 (2 doses)	S/C	2^2.5^	2^6.5^	10^6^ EID50/0.1 mL	0/9	100% *
Group 2 (1 dose)	S/C	2^4.5^	ND	10^6^ EID50/0.1 mL	3/9	70% *
Group 3 (Negative control)	ND	ND	0	ND	0/9	-
Group 4 (Positive Control)	ND	ND	0	10^6^ EID50/0.1 mL	9/9	-

* One chicken in both group 1 and group 2 was excluded from the challenge experiment due to a lack of HI titer, which could be due to technical reasons, such as missed the vaccination site, or vaccine leaked out from the injection site. ND = Not done.
